# IMHOTEP: cross-professional evaluation of a three-dimensional virtual reality system for interactive surgical operation planning, tumor board discussion and immersive training for complex liver surgery in a head-mounted display

**DOI:** 10.1007/s00464-020-08246-4

**Published:** 2021-01-21

**Authors:** Hannes Götz Kenngott, Micha Pfeiffer, Anas Amin Preukschas, Lisa Bettscheider, Philipp Anthony Wise, Martin Wagner, Stefanie Speidel, Matthias Huber, Felix Nickel, Arianeb Mehrabi, Beat Peter Müller-Stich

**Affiliations:** 1 Department of General, Visceral and Transplantation Surgery, Heidelberg University Hospital, Im Neuenheimer Feld 110, 69120 Heidelberg, Germany; 2 Institute for Anthropomatics and Robotics, Karlsruhe Institute of Technology, Karlsruhe, Germany; 3 National Center for Tumor Diseases, Dresden, Germany

**Keywords:** Virtual reality, Operation planning, Surgical training, Liver surgery, Head-mounted display, Virtual tumor board, Remote communication

## Abstract

**Background:**

Virtual reality (VR) with head-mounted displays (HMD) may improve medical training and patient care by improving display and integration of different types of information. The aim of this study was to evaluate among different healthcare professions the potential of an interactive and immersive VR environment for liver surgery that integrates all relevant patient data from different sources needed for planning and training of procedures.

**Methods:**

3D-models of the liver, other abdominal organs, vessels, and tumors of a sample patient with multiple hepatic masses were created. 3D-models, clinical patient data, and other imaging data were visualized in a dedicated VR environment with an HMD (IMHOTEP). Users could interact with the data using head movements and a computer mouse. Structures of interest could be selected and viewed individually or grouped. IMHOTEP was evaluated in the context of preoperative planning and training of liver surgery and for the potential of broader surgical application. A standardized questionnaire was voluntarily answered by four groups (students, nurses, resident and attending surgeons).

**Results:**

In the evaluation by 158 participants (57 medical students, 35 resident surgeons, 13 attending surgeons and 53 nurses), 89.9% found the VR system agreeable to work with. Participants generally agreed that complex cases in particular could be assessed better (94.3%) and faster (84.8%) with VR than with traditional 2D display methods. The highest potential was seen in student training (87.3%), resident training (84.6%), and clinical routine use (80.3%). Least potential was seen in nursing training (54.8%).

**Conclusions:**

The present study demonstrates that using VR with HMD to integrate all available patient data for the preoperative planning of hepatic resections is a viable concept. VR with HMD promises great potential to improve medical training and operation planning and thereby to achieve improvement in patient care.

**Supplementary Information:**

The online version contains supplementary material available at 10.1007/s00464-020-08246-4.

Hepatic resection is a mainstay in treatment of primary and secondary malignant hepatic lesions [1]. As per standard procedure in oncological surgery, the focus is on maximizing oncological safety and minimizing the extent of resection that goes along with functional loss. Hepatic resections can be complex surgical procedures with considerable potential for morbidity and mortality [2, 3]. High-risk procedures (extended right/left hepatectomies, central resections, polysegmentectomies, large atypical resections, repeated resections, and hepatectomies in the setting of abnormal liver parenchyma) should, therefore, be indicated and planned in a multidisciplinary team [4]. In order to decide on the most beneficial approach for each individual patient in modern multimodal treatment and surgical procedures, it is necessary to assimilate a large amount of heterogeneous data from a wide range of sources and medical disciplines [5]. Traditional ways of presenting, sharing, and interacting with this crucial information do not perfectly match the requirements for an optimal and timely decision-making process [6]. This becomes increasingly important due to the necessity to perform tumor boards and conferences at a distance and without meeting personally nowadays.

Currently, imaging data (e.g., computed tomography (CT) scan or magnetic resonance imaging (MRI)) for surgical planning and training are displayed on 2D screens in clinical routine, depriving surgeons of an authentic visualization of the intraoperative situation. Additional data and patient-related information are needed for best interpretation of imaging data. Computer-assisted surgery planning has been proven to facilitate choosing the appropriate surgical techniques in high-risk procedures and complex cases [7, 8]. By calculating pre- and postsurgical liver volume in the preoperative planning phase, the individual adequate procedure for each patient can be chosen with consideration [9]. Computer-assisted surgery planning can also assist in correctly identifying the individual patient anatomy and pathology. The feasibility and benefit of 3D preoperative liver planning in the clinical environment has already been described and commercial solutions are available. Examples are Raytracer (Heidelberg, Germany), Virtual Surgical Planning (Strasbourg, France), HepaVision (Bremen, Germany), and Synapse VINCENT (Fuji-film Medical, Tokyo, Japan) [7, 10].

In light of such recent developments in cognition-based liver planning systems, new modalities have to be explored to show and interact with preoperative and intraoperative liver planning data [11]. Available software solutions for preoperative planning do not take into account the presentation and integration of additional information such as liver function tests, altered hepatic tissue, laboratory tests, medical history and other diagnostic tests in order to decide on a treatment strategy. It is up to the interdisciplinary team to take all available data related to the individual patient into consideration [5]. Virtual reality (VR) with context sensitive presentation of information could be a viable solution for this problem. For this purpose, we developed an interactive and immersive VR system (IMHOTEP) for visualizing surgical planning data with a 3D VR framework together with all other clinical information using the commercially available head-mounted display (HMD) Oculus Rift™, as seen in Fig. 1. The technical aspect of the IMHOTEP system has been extensively discussed in a study by Pfeiffer et al. [12]. We conducted a study to evaluate the potential of the VR environment IMHOTEP to display patient information, imaging data, and 3D-models of the liver anatomy and pathology for hepatic surgery. This was chosen as a first application as it represents a potentially complex and challenging surgical procedure and will enable scalability of the platform to other surgical disciplines.

**Fig. 1:**
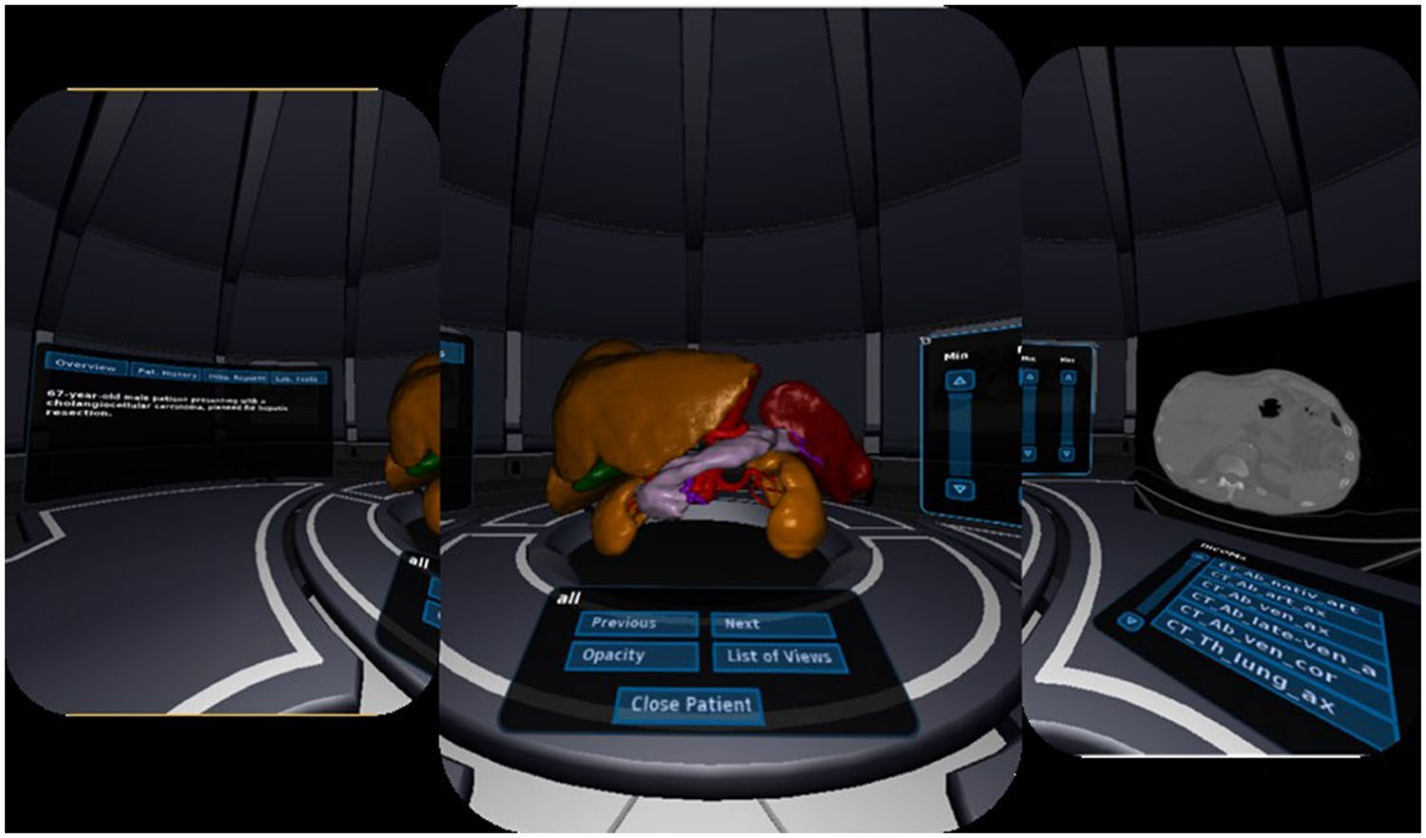
Virtual Reality environment from inside the Oculus Rift®. Patient information (left), 3D-model (middle), and original sectional imaging (right)

## Material and methods

Anonymized computed tomography (CT) images and patient information were retrieved from a patient with intrahepatic cholangiocarcinoma undergoing treatment at the Department of General, Visceral, and Transplantation Surgery at Heidelberg University Hospital. In this case, the localization of the cholangiocarcinoma based on the indication to perform an extended right hemihepatectomy. See the patient vignette (Table 1) for more detail. The patient information and data were retrieved in anonymized form from the digital patient database of the hospital. Open-source software was used for image segmentation and post-processing. The organ surfaces of the liver, kidney, pancreas, spleen, and gallbladder were segmented semi-automatically using the Medical Imaging and Interaction Toolkit (MITK, German Cancer Research Center, Heidelberg, Germany, www.mitk.org). The vessels and bile ducts were segmented semi-automatically using ITK-snap (www.itksnap.org). For the segmentations CT-images in the portal venous phase were used, with the exception of the arteries, in which case the arterial phase was used. Registration of the arterial and portal venous CT-images was done with 3D Slicer (www.slicer.org, [13]). Post-processing of the 3D-models was carried out using MeshMixer (Autodesk, San Rafael, California, U.S.A., www.meshmixer.com). Segmentations and mesh models were checked for correctness by a board-certified general surgeon and radiologist.

**Table 1:** Patient information of the visualized patient

Patient vignette	
Pathology	Intrahepatic cholangiocarcinoma
TNM	pT2a Nx M0
Stage	II
Symptoms	Painless jaundice
Past medical history	Arterial hypertension s/p hepatitis A/B glaucoma Preoperative medication: none
Past surgical history	None
Planned operation	Extended right hemihepatectomy

**Fig. 2:**
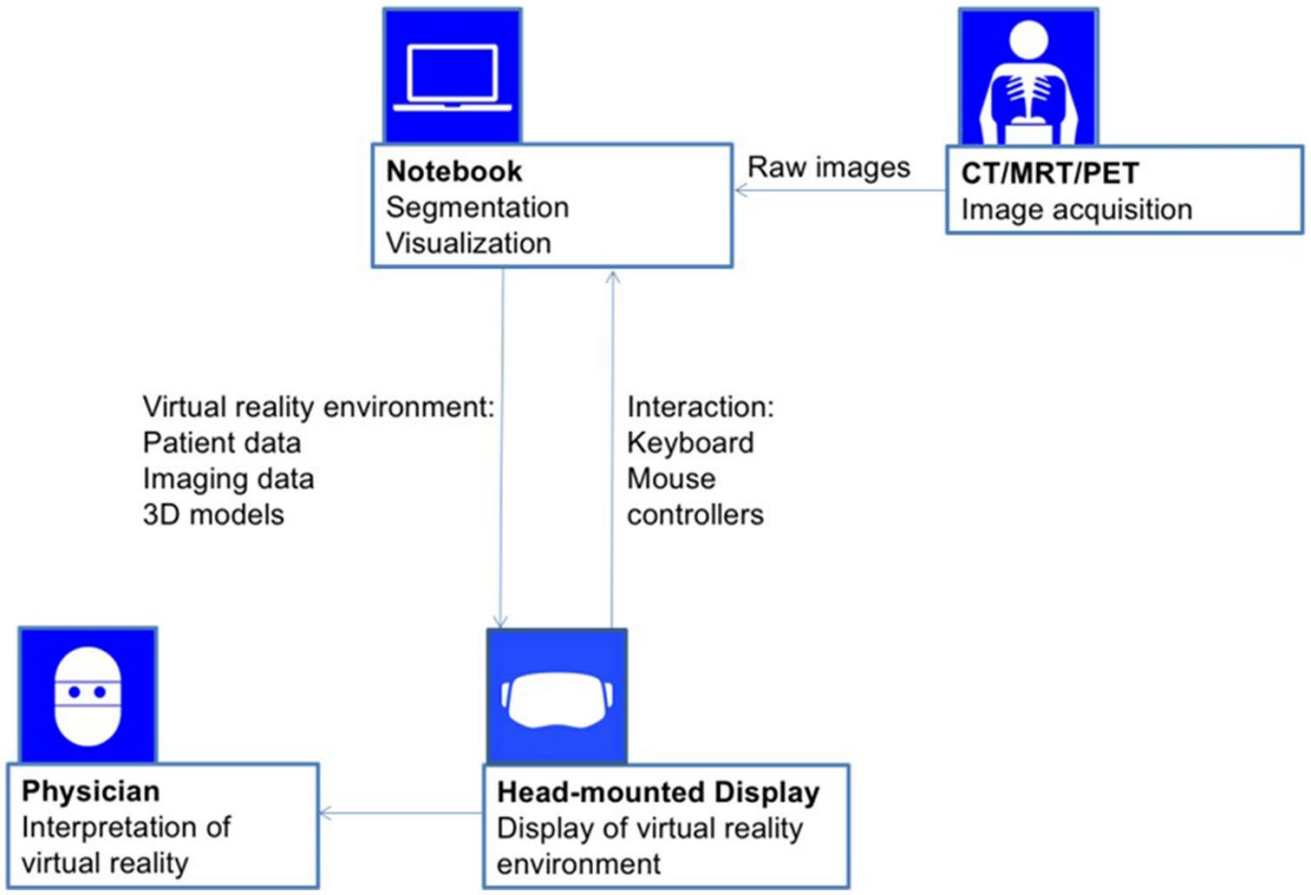
IMHOTEP virtual reality system workflow

The anonymized CT-images, patient information (patient history, diagnostic reports and laboratory results), and 3D-models were loaded into the VR software IMHOTEP, which was designed and developed together with the Karlsruhe Institute of Technology (IMHOTEP, Karlsruhe Institute for Technology, Karlsruhe, Germany, www.imhotep-medical.org). This software is a standalone feature not dependant on other hospital software, and as such is theoretically generalizable outside of the University Hospital Heidelberg. The software was installed on a laptop (XMG U505, Schenker Technologies GmbH, Leipzig, Germany) with Intel® Core™ i7-4790S CPU with 3.20 GHz, 16 GB Rapid Access Memory and NVIDIA® GeForce™ GTX 980 M graphic card. The interaction was realized with a standard computer mouse and keyboard (Fig. 2).

The immersive aspect of this study was realized by using the VR HMD Oculus Rift™ (Oculus VR LLC, Menlo Park, California, USA). The Oculus Rift™ has two LCD displays to create a stereoscopic 3D perspective. The user's head position and movement are tracked so that head movements can be translated into movement in the VR environment making the experience realistic. Figure 1 shows a panoramic screenshot of the IMHOTEP interactive virtual reality environment, Media 1 shows a clip of the HMD in use.

Participants had sufficient time to familiarize themselves with the handling of the VR environment. The clinical showcase could be evaluated for as long as the participants desired. After using the system, a 10-item online questionnaire was filled out by the participants using the Likert scale. A positive response was noted if the participant rated the question with 4 or 5 out of 5 points on the Likert scale (Table 2). The system was evaluated at the Department of General, Visceral, and Transplantation Surgery at Heidelberg University Hospital by medical students, resident surgeons, attending surgeons, and nurses. Google™ Forms (Google Inc., Mountain View, California, USA) was used for the questionnaire data acquisition.

**Table 2:** Questionnaire with median answers and interquartile ranges on the Likert scale, as well as percentage of positive responses (rating of 4 or 5)

Question	All (*n* = 158)	Medical students (*n* = 57)	Resident surgeons (*n* = 35)	Attending surgeons (*n* = 13)	Nurse (*n* = 53)	*p* value
1. Medical education background?		Student	Resident	Attending	Nurse	
2. Was the VR environment pleasant?	4 (4–5) 89.9%	4 (4–5) 91.2%	4 (4–5) 85.7%	4 (4–5) 76.9%	4 (4–5) 94.3%	0.946
3. Is the assessment of complex cases better with VR than with standard display?	5 (4–5) 94.3%	5 (4–5) 91.2%	5 (4–5) 85.7%	4 (4–5) 100%	5 (4–5) 94.3%	0.18
4. Is the assessment of complex cases faster?	4 (4–5) 84.8%	4 (4–5) 86.0%	4 (4–5) 80.0%	4 (4–5) 76.9%	5 (4–5) 88.7%	0.21
5. How highly do you rate the potential for medical student training?	4 (4–5) 87.3%	4 (4–5) 84.2%	4 (3–5) 85.7%	4 (3–5) 76.9%	4 (4–5) 94.3%	0.75
6. How highly do you rate the potential for resident training?	4 (4–5) 84.6%	4 (4–5) 85.7%	4 (4–5) 76.5%	4 (4–5) 69.2%	4 (4–5) 92.5%	0.711
7. How highly do you rate the potential for clinical use?	4 (4–5) 80.3%	4 (4–5) 87.7%	4 (4–5) 64.7%	4 (3–5) 69.2%	5 (4–5) 84.9%	**0.035**^**1**^
8. How highly do you rate the potential for nurse training?	4 (3–5) 54.8%	3 (2–4) 31.6%	3.5 (3–4.75) 50.0%	3 (2–4) 38.5%	5 (4–5) 86.8%	** < 0.001**^**2**^
9. How many years until daily clinical use? (years)	4.76	5.39	4.26	5.46	4.28	**0.04**^**3**^
10. Free comments	Various (see results)					

Significant *p* values in bold

^1^The difference in answers between nurses and medical students (*p* = 0.02), as well as nurses and residents (*p* = 0.016), were significant; ^2^The difference in answers between nurses and medical students (*p* < 0.001), nurses and residents (*p* = 0.001), and nurses and attendings (*p* = 0.001) were significant; ^3^The difference in answers between nurses and medical students (*p* = 0.003) was significant

Possible differences between groups were evaluated using a Kruskal–Wallis test. In the case of statistical significance, a two-tailed Mann–Whitney U Test was used for individual subgroup analysis. The level of significance was set to 5% (*p* ≤ 0.05). All statistical calculations were carried out using the Real Statistics Resource Pack software (Release 4.3, Copyright 2013 – 15, Charles Zaiontz, www.real-statistics.com).

IRB approval was not needed for the current study. Consent was obtained from the participants.

## Results

The system was evaluated by 158 participants consisting of 57 medical students, 35 resident surgeons, 13 attending surgeons, and 53 nurses. A majority of participants rated the VR experience as pleasant (89.9% of responses positive) and agreed that assessment of complex cases could be performed faster (84.8% of responses positive) and more comprehensively (94.3% of responses positive) when compared to conventional assessment (Table 2). All participant groups rated the training potential of this technology highly (Table 3). More than 50% of all participants rated the potential of the tool for training residents, medical students, and nursing students as well as for clinical use as positive. Nurses saw more potential of this technology for clinical application (*p* = 0.035) and for nursing training (*p* < 0.001) when compared to the other participant groups. Medical students saw significantly higher potential for the training of residents, medical student,s and clinical use than for nursing training (*p* < 0.001). No significant differences were found for the other questions; attending and resident surgeons rated both the training potential for all profession groups and the potential for clinical use of the technology positively. There were no significant differences in the answers between surgeons and medical students.

**Table 3:** Evaluation of the technology potential for training and clinical use by profession

Participants	Medical student training	Resident training	Clinical use	Nurse training	*p* value
Medical students	4 (4–5) 84.2%	4 (4–5) 85.7%	4 (4–5) 87.7%	3 (2–4) 31.6%	** < 0.001**^**1**^
Resident surgeons	4 (3–5) 85.7%	4 (4–5) 76.5%	4 (4–5) 64.7%	3.5 (3–4.75) 50%	0.14
Attending surgeons	4 (3–5) 76.9%	4 (4–5) 69.2%	4 (3–5) 69.2%	3 (2–4) 38.5%	0.093
Nurses	4 (4–5) 94.3%	4 (4–5) 92.5%	5 (4–5) 84.9%	5 (4–5) 86.8%	0.069

Answers displayed as median and interquartile ranges on the Likert scale, as well as percentage of positive responses (rating of 4 or 5). Significant *p* values in bold

^1^Medical students’ assessment of potential for nurse training differed significantly from the assessment of potential for medical student training (*p* < 0.001), resident training (*p* < 0.001), and clinical use (*p* < 0.001)

Resident surgeons and nurses expected this system to be a clinical reality within approximately four years, significantly less time than medical students and attending surgeons who expected the time to clinical use to exceed 5 years (*p* = 0.04). The evaluation of the free text comments showed that four participants (2.5%) reported motion sickness, three participants (1.9%) reported compatibility difficulties with wearing glasses, and one participant (0.6%) complained of low resolution. Twelve participants (7.6%) left additional positive comments complimenting the intuitive user interface, detailed visualization of the models, and enabling of a better understanding of the planned procedure.

## Discussion

Modern hepatic surgery is a multi-specialty surgical field. Having all the information at the appropriate time is essential to perform surgical treatment successfully, improve patients’ outcome and to reduce morbidity and mortality. In lieu of the increasing digitalization of the healthcare system and medical education, we have created an immersive 3D operation planning and training system for liver surgery using VR in a HMD to adequately aggregate and visualize patient-related data. This system attempts to integrate the 3D-models into a VR environment alongside patient information and original imaging data, optimally providing an improved understanding of complex liver operations when compared to current practice, i.e., 2D-visualization on a computer screen. This assumption is underlined by the results that the majority of the participants in the present study, including experienced surgeons, found the VR environment pleasant and agreed that complex cases could be assessed better and faster with this technology than with traditional methods. Additionally, the broad multidisciplinary appeal of this technology combined with the positive answers regarding clinical feasibility across the entire study population is an indicator that this or similar software could find broad application when discussing individualized procedures in an interdisciplinary team, such as improving data visualization during conferences or on virtual tumor board reviews. Especially in times when tumor boards or other meetings have to be performed at a distance this can prove very useful. In the case of the patient data and lab values, these are currently loaded manually into the IMHOTEP software. An integration with existing digital healthcare technology, such as digital patient database or the hospital’s image viewer software could be a potential further step in improving the technological compatibility. The current limiting factor of the IMHOTEP system, however, is not the time required for manual loading of patient data, but rather the expertise and time required to produce 3D segmentations of anatomical structures from existing CT or MRI images. This is discussed further in the limitations section.

New technologies are becoming increasingly adopted in healthcare, e.g., using tablet computers, electronic health care records and 3D animations. Computer-assisted surgery planning has already been evaluated and proven beneficial for high-risk hepatectomy cases, central tumors, and living donor liver transplantations [7, 11, 14, 15]. Virtual hepatic resections aid in determining the remnant and resection volumes as well as in the anticipation of crucial intraoperative steps based on the patient-specific anatomy [9, 14].

Considerable potential for IMHOTEP was also seen in student and resident training, with student training being considered as having the highest potential in the present study. Already, the use of 3D-models and 3D presentations for teaching has been the subject of several studies [16, 17]. Barsom et al. encouraged the use of new VR and AR technologies to open new horizons in medical education [18]. We argue that the IMHOTEP system may facilitate the teaching of liver anatomy and pathology and could help medical students to understand the steps in deciding on a hepatic resection technique in surgery [16]. This could be achieved for example by providing multiple patient cases and a step-by-step analysis of how their respective procedure was chosen from the available data. Müller-Stich et al. already showed that 3D-visualizations of the liver improved the identification of hepatic anatomy [19] as compared to 2D-visualization. Accordingly, Jurgaitis et al. showed that with 3D visualization medical students’ ability to localize hepatic tumors and to determine the extent of the hepatic resection was improved as compared to 2D [20].

Resident surgeons may also benefit from VR HMD systems. By having all required information for making a surgical decision easily accessible, a greater understanding of how and why certain decisions are made could be achieved. Certain published studies also show growing interest in using these tools to train residents [21]. As surgical training outside of the direct operative setting has always been a major consideration for training programs, considerable effort is being invested to evaluate the possibilities of modern technology for the education of surgical trainees. As examples, Roberts et al. describes that surgical simulation using VR can help make surgical training outside the operating room more realistic [22]. Nickel et al. described positive results for training new surgical procedures to surgical trainees in a virtual environment as well as in a digital learning setting [17, 23]. The 3D Definite Human is a project of the Royal college of Surgeons of Edinburgh to teach surgical trainees surgical anatomy and interventions based on 3D-models [24]. In a systematic review by Graafland et al. of 27 studies, serious games were shown to be used for training of technical and non-technical skill training and harbor the potential to improve the education of complex decision-making processes in surgery. Advantages are seen for understanding different situations as well as the opportunity of a contextual and more realistic learning experience [25].

The current study showed that attending surgeons responded positively and showed a marked interest in VR visualization of complex operations. Preoperative simulation of the patient-specific anatomy and pathology may be beneficial to anticipate critical situations in the OR and might also increase situational awareness [26, 27]. Complex liver surgery is mostly performed at specialized centers by experts in hepatic surgery who can often anticipate operative steps with the aid of MRI- or CT-images, it must also be considered that hepatic surgery is becoming increasingly complex and indications are being widened for individual procedures. This leads to more information needing to be present and processed by the operating surgeon at any single moment [28]. The well experienced attending surgeons participating in this study answered that assessment of complex cases could be improved both in quality (100% positive response) and time required (76% positive response) by using VR with HMD.

Health care professionals saw least potential of the VR and HMD technology in nursing training, although nurses themselves saw high potential of the technology in nursing training (92.5% positive response). One possible explanation for the difference is that nurses may be eager to learn more about the operations that they are playing an integral part in and thus find this technology useful. Johnston et al. reported that for nursing students, the anatomy courses are very content heavy and sometimes challenging [29]. A VR environment may facilitate the understanding of the human anatomy and surgical decision-making, thus potentially improving this area. Glaser et al. presented an interactive training system for scrub nurses to simulate a live operating room situation outside the operating room. Such a tool might help to prepare scrub nurses better, in particular novices, and increase understanding of operative procedures [30]. It is certainly worth considering the potential applications in VR regarding nursing training, and it can be argued that the current study makes a good case for the nursing profession having a strong interest in seeing these technologies being applied in their field.

As this manuscript is an analysis of the professional evaluation of potential applicability, there are no measurable surgical endpoints to report. The current study was not designed to evaluate the effectiveness of the technology in a clinical setting, but rather to gauge the response of healthcare professionals to the potential use and applicability of the IMHOTEP system. This favorable response can form the basis of further evaluations of this integrated system, such as case evaluation of complex surgeries by novices and professionals with measurable endpoints such as time to response, and finally potential pilot trials in the real clinical setting. In this context, barriers to expansion that must be considered are for one the difficulty of technological adaptation and the sensitive topic of sharing patient data online. There have been multiple studies looking at the difficulty of implementing VR and mixed reality technology in other fields, and willingness of adoption varies strongly between individuals [31, 32]. As such, and despite the generally positive response of the participants in the current study, a critical evaluation and re-evaluation of user friendliness and graphical user interface (GUI) changes during further development of the IMHOTEP system must be prioritized. On the side of ethical data management and patient safety, implementation of online interaction with other hospitals must be kept secure when potentially implementing VR, for example in clinical situations such as tumor boards or similar multidisciplinary conferences. The ethics of online patient data or hospital implementations of cloud computing is complex, and a full discussion of risks and benefits of this technology would be far out of the scope of the current manuscript. Fortunately, Implementation of e.g., a virtual tumor board could orient itself along existing clinical information sharing systems that have been established and in use for many years [33, 34]. Should this technology be shown as feasible and useful in the further development and clinical application, expansion and reproduction in other centers would be desired.

## Limitations

Upon review of the methods used in the current study, it should be noted that the questionnaire could be interpreted as having a positive leading bias, as known potential negative aspects of the VR system such as motion sickness and individual compatibility were only evaluated in free text comments, and not in the Likert scale method. This should be noted as a weakness of the study design. A small number of participants (*n* = 4, 2.5%) reported motion sickness while in the VR environment. This issue is an active area of research and better devices (with smaller latency and higher resolutions) will most likely further reduce this side effect. Otherwise it is assumed that irritations could be reduced through frequent use of such devices. In the current study, participants were recruited from the staff at Heidelberg University Hospital. The choice for this recruitment was made because of the local availability of the multiple professions that the study wished to evaluate. Nevertheless, a potential favorable bias due to pure internal feedback should be seen as a limitation in this study. However, the questionnaire answers were anonymized fully, which should be argued as counteracting the potential bias that may occur in an internal evaluation, as answers could not be traced back to individual participants. Furthermore, none of the participants were involved in the conception, design, or analysis of either the IMHOTEP system or the study itself. To eliminate any remaining bias, a further step for the development of the IMHOTEP system should incorporate a similar evaluation as the one conducted in the current study by an external participant group. A further limitation that must be acknowledged is the use of only a single patient and segmentation for the evaluation of the technology. However, the significantly positive response should be seen as an argument that this case was understandable in the VR setting. Considering the complexity of liver anatomy and the difficulty of resection planning, achieving a favorable response in this patient case should be seen as a positive argument for the technology. As a final limitation, the segmentation process to provide exact 3D-models currently takes a considerable amount of manual processing time which might be a limiting factor in the wide application of this technology and is certainly a limiting factor when considering current clinical application. It is expected that with growing experience and technological advancements in automatic segmentation and registration software this problem will likely be overcome for future routine use of IMHOTEP. In the current study the interaction with the Oculus Rift™ was done using standard input devices such as a mouse and keyboard. The commercial version of the newer Oculus Rift and other HMDs are shipped with motion sensitive gesture control for a more intuitive interaction in the VR environment [35] (https://www.oculus.com/).

## Conclusion

The IMHOTEP system uses a VR environment with a HMD for immersive surgical planning and training of complex hepatic operations with three-dimensional display of imaging and integration of all necessary patient information. The use of the developed system was feasible and found approval by the majority of medical personnel. Medical professionals and students saw potential for this technology in student and resident training as well as in clinical use. Nursing professionals saw high potential for this technology in nursing training. The next step will be to evaluate its concrete improvement of training and operation planning and the scalability to other operation techniques as well as the implementation in the curriculum. Great potential for virtual tumor boards and conferences is seen especially in times where personal meetings are restricted or impossible.

## Supplementary Information

Below is the link to the electronic supplementary material.

Supplementary file1 (MP4 28948 KB)
